# Comparison Between the Acurate Neo and the Acurate Neo 2 Transcatheter Heart Valve System: Impact of Midframe Under‐Expansion

**DOI:** 10.1002/ccd.70839

**Published:** 2026-09-01

**Authors:** Lisa Crusius, Thaddäus Ast, Stephan Haussig, Jean‐Honoré Steul, Felix J. Woitek, Peter Sick, Tomas Madej, Utz Kappert, Axel Linke, Norman Mangner

**Affiliations:** ^1^ Department of Internal Medicine and Cardiology Heart Center Dresden, Technische Universität Dresden Dresden Germany; ^2^ Department of Cardiac Surgery Heart Center Dresden, Technische Universität Dresden Dresden Germany

**Keywords:** acurate, paravalvular leakage, TAVI, under‐expansion

## Abstract

**Background:**

Transcatheter aortic valve implantation (TAVI) with the Acurate Neo has been linked to a significant incidence of paravalvular leakage (PVL), addressed by the Acurate Neo 2 incorporating an active sealing skirt. Recent findings suggest that midframe under‐expansion is associated with worse outcomes.

**Aims:**

We investigated the hemodynamic and clinical outcomes after TAVI with Acurate Neo 2 compared to Acurate Neo and assessed the impact of midframe under‐expansion.

**Methods:**

Patients who received Acurate Neo (*n* = 124) or Acurate Neo 2 (*n* = 133) between February 2018 and December 2021 (switch to Acurate Neo 2 in November 2020) were included. The primary outcomes were VARC‐3 defined device success and early safety.

**Results:**

Device success was higher with Acurate Neo 2 compared with Acurate Neo (94.7% vs. 86.3%, *p* = 0.020), as was early safety (87.2% vs. 74.2%, *p* = 0.008). The rate of mild/moderate PVL was significantly lower in Acurate Neo 2. Kaplan−Meier estimated 1‐year survival (Acurate Neo 2: 87.8% vs. Acurate Neo: 89.0, *p* = 0.930) and freedom from the combined clinical endpoint death, stroke, myocardial infarction, and heart failure rehospitalization at 1 year (Acurate Neo 2: 81.2% vs. Acurate Neo: 79.3%, *p* = 0.528) were comparable between groups. A long‐term analysis of these endpoints revealed no difference between groups. Midframe under‐expansion occurred in 13.3% and was associated with lower freedom from the combined clinical endpoint at 1 year, irrespective of prosthesis generation.

**Conclusion:**

Acurate Neo 2 demonstrated significant improvements in device success and early safety compared to Acurate Neo. Midframe under‐expansion occurred with both generations and was associated with worse clinical outcomes within the first year.

## 1 Introduction

Over the past two decades, transcatheter aortic valve implantation (TAVI) has become standard practice for a large group of patients with severe aortic stenosis, beginning with inoperable patients [1]. The indication has expanded to include younger and low‐risk patients [2, 3, 4, 5].

This was made possible by the growing experience of operators [6], as well as advancements in valve design and the engineering of valve types that address issues such as paravalvular leakage (PVL), patient‐prosthesis mismatch, and the need for permanent pacemaker implantation (PPI). These factors have been identified as drivers of long‐term mortality [7, 8, 9].

The Acurate TF heart valve [10] and its successor the Acurate Neo valve (BostonScientific, Marlborough, MA, USA) [11] were specifically designed to mitigate the risks of PVL and PPI by its supra‐annular design with an upper crown for stable positioning and minimal LVOT protrusion of the lower crown.

The randomized Scope I and Scope II trials failed to demonstrate the noninferiority of the Acurate Neo valve, as defined by the composite endpoint, compared to other commercially available valves, such as the Sapien 3 or Evolut R/Pro. The results were primarily influenced by higher rates of PVL [12, 13].

Accordingly, the next‐generation Acurate Neo 2 valve design featured an active sealing mechanism that reduces PVL rates by enlarging the sealing skirt by 60% [14]. The Acurate Neo 2 was evaluated in the ACURATE IDE trial, in which it did not meet noninferiority. The Acurate Neo 2 resulted in significantly worse outcomes compared with commercial valves with respect to the primary endpoint of a composite of all‐cause mortality, all‐stroke, and rehospitalization at 1 year [15]. A post hoc analysis revealed that under‐expansion of the Acurate Neo 2 valve in the ACURATE IDE study was associated with a higher risk of the composite primary endpoint [16].

This analysis aims to provide real‐world experience using the Acurate Neo 2 device compared to its predecessor, with regard to early safety and efficacy as defined by VARC‐3 criteria, as well as 1‐year and 3‐year outcomes. Additionally, this analysis examines the impact of midframe under‐expansion on clinical outcomes with both iterations.

## 2 Methods

### 2.1 Study Design

This analysis includes consecutive patients with symptomatic severe aortic stenosis who underwent transfemoral valve implantation with the Acurate Neo and Acurate Neo 2 prosthesis at the Heart Center Dresden, Germany, from February 2018 to December 2021, with multidisciplinary heart team consensus for the procedure.

The implantation of the newer‐generation Acurate Neo 2 valve began in November 2020, after which this device was used for all subsequent cases. Patients provided informed written consent before the procedure. Baseline characteristics, pre‐procedural imaging data, and procedural and outcome data were collected prospectively. Follow‐up was performed at 30 days, 1 year, and 3 years. This study complies with the Declaration of Helsinki, and the local ethics committee approved data acquisition (EK 41012019). The data that support the findings of this study are available from the corresponding author upon reasonable request. None of the contents of this manuscript have been produced by artificial intelligence.

### 2.2 Device Details

The Acurate Neo 2 valve was a self‐expanding, transcatheter heart valve with a supra‐annular design. Its nitinol frame features axial stabilization, arches, and porcine pericardium leaflets. To reduce rates of paravalvular leak (PVL) due to the irregular anatomy of calcified native valves, a porcine pericardium sealing skirt had been integrated, and its height has been enlarged by 60% compared to the previous generation of the Acurate Neo. This provided synchronous adaptation in different phases of the cardiac cycle and active sealing.

The Acurate Neo 2 prosthesis was available in three sizes: S (annulus diameter: 21−23 mm), M (annulus diameter: 23−25 mm), and L (annulus diameter: 25−27 mm). Implantation was performed via a dedicated 14 F transfemoral delivery system in most of the cases (iSleeve, Boston Scientific).

### 2.3 Clinical Outcomes

Clinical outcomes were collected and analyzed according to the VARC‐3 recommendations [17]. The recommendations define technical success as freedom from mortality, successful implantation of a single prosthetic heart valve in the proper anatomical location, and freedom from surgery or intervention related to the device or a complication. This is evaluated immediately after the procedure. According to VARC 3, device success is a composite evaluated 30 days after the procedure. It consists of technical success; freedom from mortality, surgery, or intervention related to the device or a major complication; and intended valve performance, which is defined as a mean gradient of less than 20 mmHg, a peak velocity of less than 3 m/s, a Doppler velocity index of greater than 0.25, and less than moderate aortic regurgitation. The early safety endpoint after 30 days consists of freedom from all‐cause mortality, as well as freedom from all stroke, VARC type 2–4 bleeding, major vascular or access‐related complications, acute kidney injury stage 3 or 4, moderate or severe aortic regurgitation, and new PPI.

Clinical evaluations for freedom from all‐cause mortality and the composite clinical outcome of freedom from all‐cause mortality, stroke, myocardial infarction, and heart failure rehospitalization were performed at discharge, 30 days, and within 1‐year and 3‐years of follow‐up. Echocardiography was performed at discharge and at follow‐up for patients who visited our center.

### 2.4 Definition of Midframe Under‐Expansion

Midframe under‐expansion was defined as the presence of non‐parallelism of the commissure posts (main criterium) applying the boundary method as previously described [16]. It was assessed in two different projections, after final deployment and postdilatation, if performed. Additionally, the following two factors were considered in borderline cases: (1) the prosthesis being taller than its width at the level of the upper crown; and/or (2) the inflow being wider than the upper crown. An inter‐ and intraobserver variability for this approach tested in 30 randomly selected cases showed good agreement as indicated by a Cohen's kappa‐coefficient of 0.87 (95% confidence interval (CI) 0.48; 1.0) for both inter‐ and intraobserver variability.

### 2.5 Statistical Analysis

Statistical analysis was performed using SPSS software version 29.0 (IBM Corporation, Armonk, NY, USA). A *p* value of < 0.05 was considered significant. Categorical variables are expressed as numbers and percentages and were compared with the use of the chi‐squared test or Fisher's exact test, as appropriate. Continuous variables are expressed as the median with corresponding 25th and 75th percentile after testing for normal distribution applying the Shapiro−Wilk test. The analysis for freedom from all‐cause mortality and for freedom from the composite clinical outcome was performed by applying the method of Kaplan−Meier, and group comparisons were made by the log‐rank test. We derived STS‐score‐adjusted hazard ratios (HR) and their corresponding 95% CI from a Cox regression analysis.

Factors showing a *p* < 0.05 in univariate analysis were included in a multivariable binary logistic regression model to evaluate characteristics associated with device success, early safety, PVL, and midframe under‐expansion. Values are given as odds ratios (OR) and corresponding 95% CI.

A Cox regression analysis was conducted to derive HR and 95% CI for comparing midframe under‐expansion versus no midframe under‐expansion with regard to freedom from the composite clinical outcome adjusted for STS‐score and prosthesis generation.

## 3 Results

### 3.1 Baseline Characteristics

A total of 257 patients treated between 02/18 and 12/21 were included in this retrospective analysis. Treatment with the Acurate Neo and the Acurate Neo 2 was performed in 124 (48.2%) and 133 (51.8%) patients. The baseline characteristics of the two groups were comparable with regard to age and sex (Table 1). There were significant differences in the prevalence of comorbidities (atrial fibrillation, peripheral artery disease, diabetes, prior coronary artery bypass grafting) leading to a higher perioperative risk in Acurate Neo compared to Acurate Neo 2 as indicated by STS‐scores of 4.4% versus 3.3% (*p* < 0.001) (Table 1).

**Table 1:** Baseline and procedural characteristics.

	Acurate neo (*n* = 124)	Acurate neo 2 (*n* = 133)	*p* value
Baseline			
Age [years]	83 (80; 87)	83 (81; 86)	0.868
Male gender, *n* (%)	41/124 (33.1)	49/133 (36.8)	0.526
Body mass index [kg/m^2^]	26.2 (24.1; 29.6)	27.3 (23.8; 30.4)	0.429
STS‐Score [%]	4.4 (2.9; 7.0)	3.3 (2.5; 4.3)	< 0.001
NYHA III/IV, *n* (%)	68/121 (56.2)	78/131 (59.5)	0.591
CAD, *n* (%)	74/124 (59.7)	74/133 (55.6)	0.513
Previous CABG, *n* (%)	17/124 (13.7)	8/133 (6.0)	0.038
Prev. PCI, *n* (%)	20/124 (16.1)	17/133 (12.8)	0.445
Atrial fibrillation, *n* (%)	52/142 (41.9)	31/133 (23.3)	0.001
Arterial hypertension, *n* (%)	119/124 (96.0)	126/133 (94.7)	0.640
Diabetes mellitus, *n* (%)	53/124 (42.7)	41/133 (30.8)	0.048
Prev. stroke, *n* (%)	10/124 (8.1)	13/133 (9.8)	0.631
PAD, *n* (%)	18/124 (14.5)	5/133 (3.8)	0.003
COPD, *n* (%)	18/124 (14.5)	10/133 (7.5)	0.072
CKD stage ≥ 3b, *n* (%)	49/121 (40.5)	43/133 (32.2)	0.176
Immunosuppressive therapy, *n* (%)	9/124 (7.3)	6/133 (4.5)	0.348
Echocardiography			
LV‐ejection fraction [%]	59 (50; 65) *N* = 121	57 (49; 64) *N* = 129	0.528
Aortic valve area [cm^2^]	0.7 (0.6; 0.9) *N* = 123	0.7 (0.6; 0.8) *N* = 130	0.370
Mean gradient [mmHg]	39 (26; 46) *N* = 120	39 (29; 46) *N* = 130	0.560
Mitral regurgitation 2/3, *n* (%)	34/124 (27.4)	22/129 (17.1)	0.047
Computed tomography			
Perimeter derived annulus [mm]	23.2 (22.0; 24.6)	23.2 (22.1; 24.5)	0.746
Perimeter‐derived LVOT [mm]	23.6 (22.2; 25.1)	23.4 (22.1; 25.2)	0.756
Perimeter derived STJ [mm]	27.7 (25.9; 29.3)	27.2 (25.6; 29.5)	0.609
Height STJ [mm]	21.3 (19.9; 23.0)	21.9 (20.2; 23.1)	0.143
Height LCA [mm]	13.1 (11.3; 15.2)	13.2 (11.4; 15.3)	0.993
Height RCA [mm]	17.0 (15.2; 18.5)	16.5 (15.0; 18.2)	0.255
Eccentricity annulus	1.32 (1.26; 1.40)	1.32 (1.26; 1.39)	0.825
Cover‐index [%]	7.8 (4.8; 10.0)	7.2 (4.4; 9.6)	0.589
Calcium score DLZ [AU]	2562 (866; 6613)	2496 (608; 6124)	0.511
Calcium score leaflets [AU]	2333 (818; 5720)	2386 (595; 5062)	0.511
Calcium score LVOT [AU]	151 (10; 816)	144 (10; 844)	0.713
Cusp symmetry, *n* (%)	63/124 (50.8)	59/133 (44.4)	0.206
CFA at access site [mm]	8.3 (7.5; 9.0)	8.1 (7.5; 8.8)	0.778
Moderate/severe calcification CFA, *n* (%)	28/123 (22.8)	39/132 (29.5)	0.219
Procedure			
Indication			
Native valve	124/124 (100)	132/133 (99.2)	1.000
Valve‐in‐valve	0/124 (0)	1/123 (0.8)	
Valve size			0.820
Large (27 mm)	42/124 (33.9)	43/133 (32.3)	
Medium (25 mm)	50/124 (40.3)	51/133 (38.3)	
Small (23 mm)	32/124 (25.8)	39/133 (29.3)	
Predilatation, *n* (%)	120/124 (96.8)	129/132 (97.7)	0.715
Predilatation undersizing, [mm]	1.1 (0.5; 1.7)	1.4 (1.0; 2.1)	< 0.001
Postdilatation, *n* (%)	94/123 (76.4)	83/133 (62.4)	0.015
Postdilatation undersizing, [mm]	0.7 (−0.1; 1.7)	1.2 (0.8; 2.0)	< 0.001

*Note:* Variables are expressed as numbers and frequencies or median with 25th and 75th quartile.

Abbreviations: CABG, coronary artery bypass surgery; CAD, coronary artery disease; CFA, common femoral artery; CKD, chronic kidney disease; COPD, chronic obstructive lung disease; DLZ, device landing zone; LCA, left coronary artery; LV, left ventricular; LVOT, left ventricular outflow tract; NYHA, New York Heart Association; PAD, peripheral artery disease; PCI, percutaneous coronary artery intervention; RCA, right coronary artery; STJ, sinotubular junction; STS, Society of Thoracic Surgery.

Pre‐implantation echocardiographic findings did not differ significantly between the two groups except for higher rates of moderate to severe concomitant mitral regurgitation in Acurate Neo compared to Acurate Neo 2 (Table 1).

The median CT‐derived calcium score was 2562 AU in the Acurate Neo and 2496 AU in the Acurate Neo 2 group (*p* = 0.511) indicating severely calcified valves in a population with two thirds of female patients. Other CT‐derived parameters were not different between groups (Table 1).

### 3.2 Procedural Characteristics

Both groups showed similar procedural characteristics (Table 1). Valve sizes were evenly distributed between groups. Predilatation was performed in the majority of patients in both the Acurate Neo (96.8%) and Acurate Neo 2 (97.7%). Postdilatation rates were higher with the Acurate Neo than with the Acurate Neo 2 (76.4% vs. 62.4%, *p* = 0.015), although undersizing of the balloon was higher with the Acurate Neo 2 than with the Acurate Neo for both pre‐ and postdilatation.

### 3.3 Procedural Outcomes

Outcomes defined by VARC 3 endpoints are shown in Figure 1. Technical success was achieved in 96.8% and 97.7% in the Acurate Neo and the Acurate Neo 2 group, respectively.

**Figure 1:**
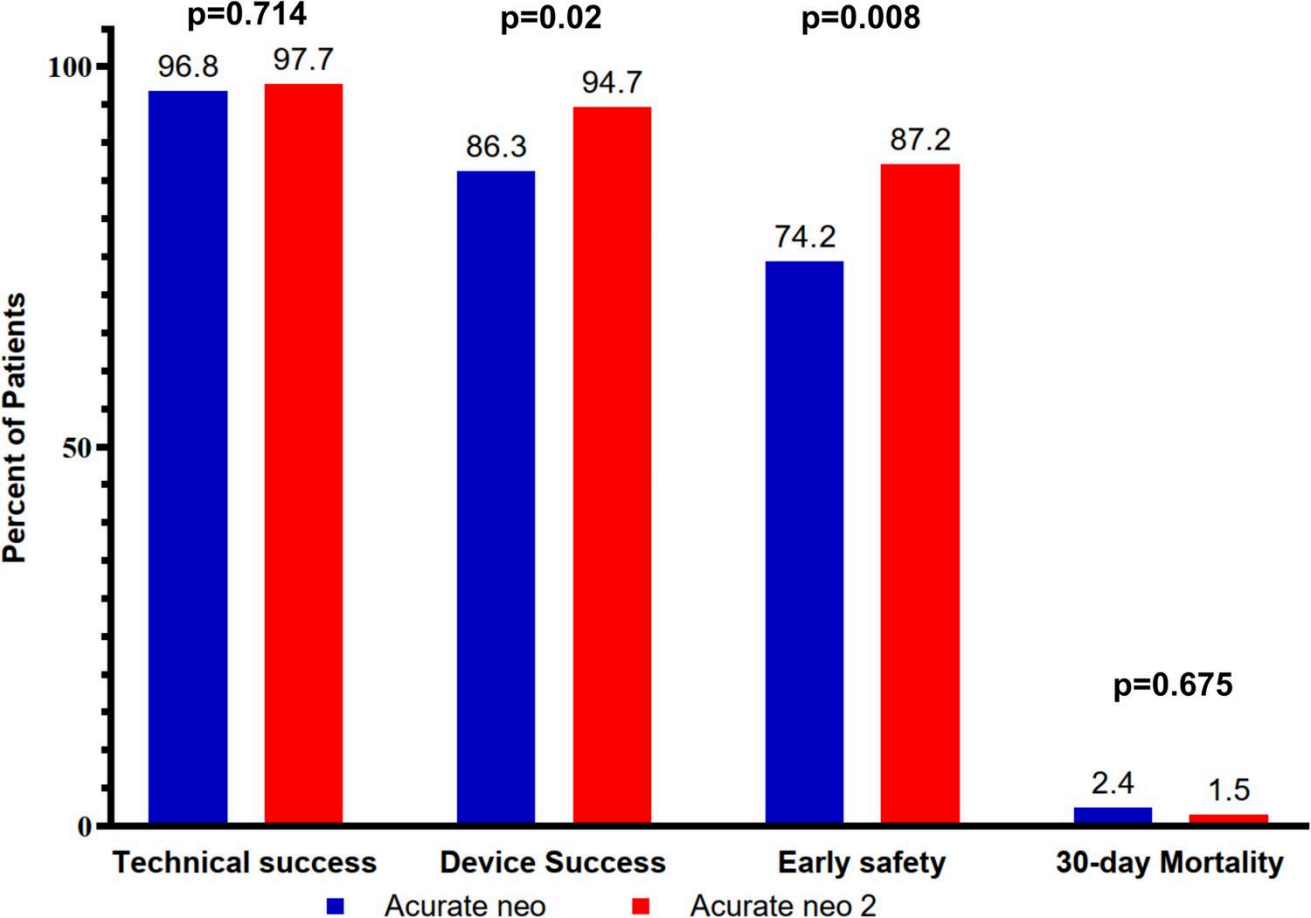
VARC‐3‐defined composite outcomes and 30‐day mortality comparing Acurate Neo with Acurate Neo 2. [Color figure can be viewed at wileyonlinelibrary.com]

Acurate Neo 2 showed significantly higher device success (94.7% vs. 86.3%, *p* = 0.020) and early safety (87.2% vs. 74.2%, *p* = 0.008). Mortality at 30 days was low and similar between groups (1.5% for Acurate Neo 2 vs. 2.4% for Acurate Neo, *p* = 0.675). Life‐threatening or major bleeding events were less frequent in Acurate Neo 2 (3.0% vs. 8.9%, *p* = 0.045). Other major complications, such as stroke, access site complications, acute kidney injury, and new PPI, were comparable between the two groups (Table 2).

**Table 2:** Procedural outcome.

	Acurate neo (*n* = 124)	Acurate neo 2 (*n* = 133)	*p* value
Technical success, *n* (%)	120/124 (96.8)	130/133 (97.7)	0.714
Device success, *n* (%)	107/124 (86.3)	126/133 (94.7)	0.020
Early safety, *n* (%)	92/124 (74.2)	116/133 (87.2)	0.008
30‐day mortality, *n* (%)	3/124 (2.4)	2/133 (1.5)	0.675
Myocardial infarction, *n* (%)	0/113 (0)	1/133 (0.8)	1.000
Stroke, *n* (%)	3/114 (2.6)	3/133 (2.3)	1.000
Life‐threatening/major bleeding, *n* (%)	11/124 (8.9)	4/133 (3.0)	0.045
Major access site complication, *n* (%)	4/124 (3.2)	2/133 (1.5)	0.433
Renal failure, *n* (%)	15/124 (12.1)	12/133 (9.0)	0.422
New PPI/ICD, *n* (%)	11/96 (11.5)	8/114 (7.0)	0.264
Residual AR			0.004
No AR	30/124 (24.2)	54/132 (40.9)	
Mild AR	88/124 (71.0)	77/132 (58.3)	
Moderate AR	6/124 (4.8)	1/132 (0.8)	
Residual mean gradient [mmHg]	7 (5; 9)	7 (5; 9)	0.994

*Note:* Variables are expressed as numbers and frequencies or median with 25th and 75th quartile.

Abbreviations: AR, aortic regurgitation; ICD, implantable cardioverter defibrillator; PPM, permanent pacemaker.

Multivariable logistic regression analysis identified the use of the Acurate Neo 2 valve as an independent predictor of device success (OR 2.68, 95% CI 1.04; 6.94) and early safety (OR 2.36, 95% CI 1.20; 4.65). Additional predictors are shown in Supporting Information S1: Tables 1 and 2.

### 3.4 Echocardiographic and Symptomatic Outcomes

Hemodynamic performance, as measured by residual mean gradient, was similar in both groups, with a median residual gradient of 7 mmHg in both Acurate Neo 2 and Acurate Neo at discharge (*p* = 0.994), remaining stable at follow‐up (Figure 2).

**Figure 2:**
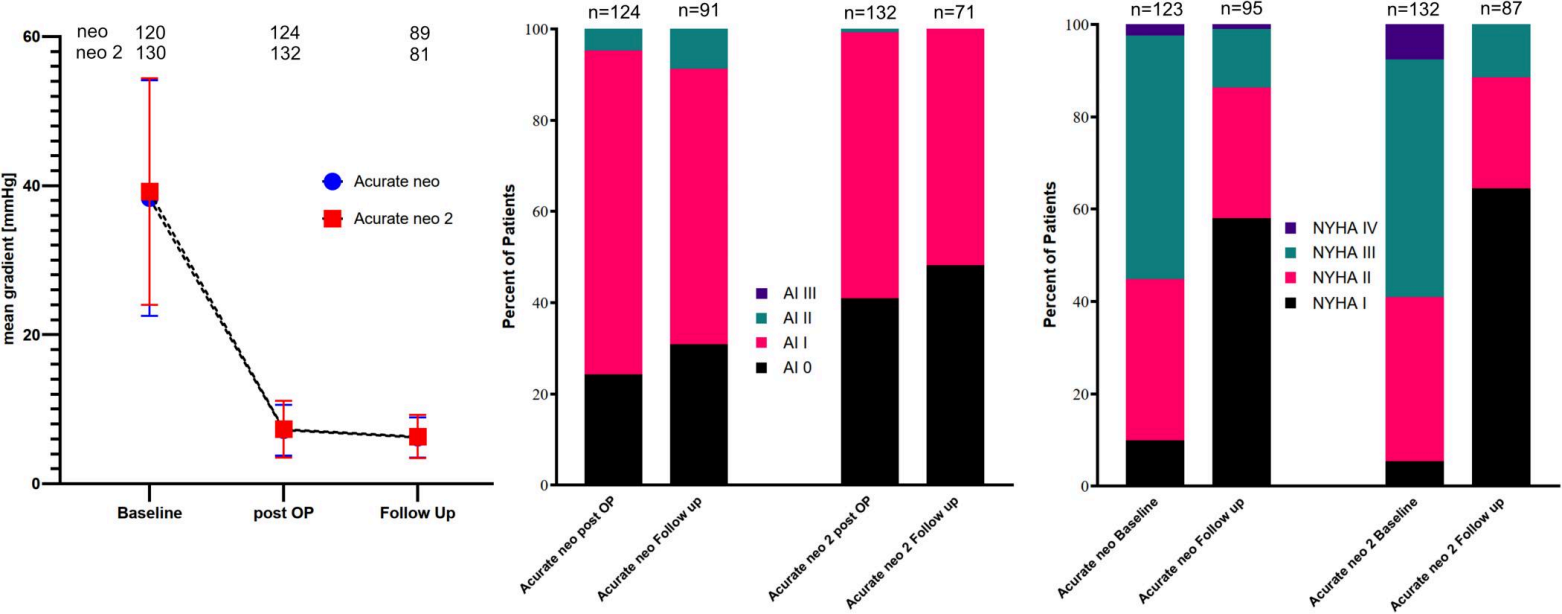
Hemodynamic and symptomatic outcome comparing Acurate Neo with Acurate Neo 2. [Color figure can be viewed at wileyonlinelibrary.com]

The Acurate Neo 2 showed significantly lower rates of residual PVL compared to the Acurate Neo (Figure 2). At discharge, no AR was observed in 40.9% of Acurate Neo 2 compared to 24.2% in the Acurate Neo. Mild AR was less frequent in Acurate Neo 2 (58.3% vs. 71.0%), and moderate AR was almost eliminated in the Neo 2 group (0.8% vs. 4.8%). This finding remained similar at follow‐up (Figure 2). The use of the Acurate Neo 2 valve was associated with a significantly lower likelihood of any PVL (OR 0.47, 95% CI 0.27; 0.81). Higher calcification in the device landing zone was also predictive of increased PVL (OR 1.01 per 100 AU increase, 95% CI 1.00; 1.02) (Table 3).

**Table 3:** Predictors of any paravalvular regurgitation.

	Univariate	Multivariable		
	OR (95% CI)	*p* value	OR (95% CI)	*p* value
Acurate neo 2 versus neo	0.46 (0.27; 0.79)	0.005	0.47 (0.27; 0.81)	0.006
Age [years]	1.04 (0.98; 1.11)	0.195		
Male gender, *n* (%)	0.83 (0.48; 1.42)	0.492		
LV‐ejection fraction [%]	0.99 (0.97; 1.01)	0.437		
Mean gradient [mmHg]	1.00 (0.98; 1.02)	0.801		
Mitral regurgitation 2/3, *n* (%)	0.87 (0.46; 1.64)	0.666		
Valve size				
Large (27 mm)	reference			
Medium (25 mm)	0.95 (0.51; 1.77)	0.872		
Small (23 mm)	0.75 (0.38; 1.46)	0.390		
Predilatation, *n* (%)	0.81 (0.15; 4.26)	0.803		
Postdilatation, *n* (%)	1.25 (0.74; 2.12)	0.405		
Perimeter derived annulus [mm]	1.12 (0.97; 1.30)	0.115		
Eccentricity index	1.01 (1.00; 1.02)	0.369		
Cover‐index [%]	0.94 (0.88; 1.01)	0.093		
Calcium score DLZ > versus < median	1.47 (0.87; 2.49)	0.155		
Cusp calcification (per 100 AU increase)^a^	1.01 (1.00; 1.02)	0.019		
LVOT Calcification (per 100 AU increase)^a^	1.05 (0.99; 1.11)	0.112		
DLZ Calcification (per 100 AU increase)	1.01 (1.00; 1.02)	0.024	1.01 (1.00; 1.02)	0.025

^a^ Not included to avoid collinearity.

Symptoms improved in both groups with 86% of patients in Acurate Neo and 89% of patients in Acurate Neo 2 reporting NYHA I/II symptoms at follow‐up (Figure 2).

### 3.5 Clinical Outcomes at 1‐ and 3‐Year Follow‐Up

The Kaplan−Meier estimated 1‐year survival (Acurate Neo 2: 87.8% [95% CI 79.8; 96.7] vs. Acurate Neo: 89.0% [95% CI 82.9; 95.5], *p* = 0.930) was comparable between groups. The STS‐score‐adjusted HR for 1‐year survival comparing Acurate Neo 2 versus Acurate Neo was 1.19 (95% CI 0.46; 3.09) (Figure 3).

**Figure 3:**
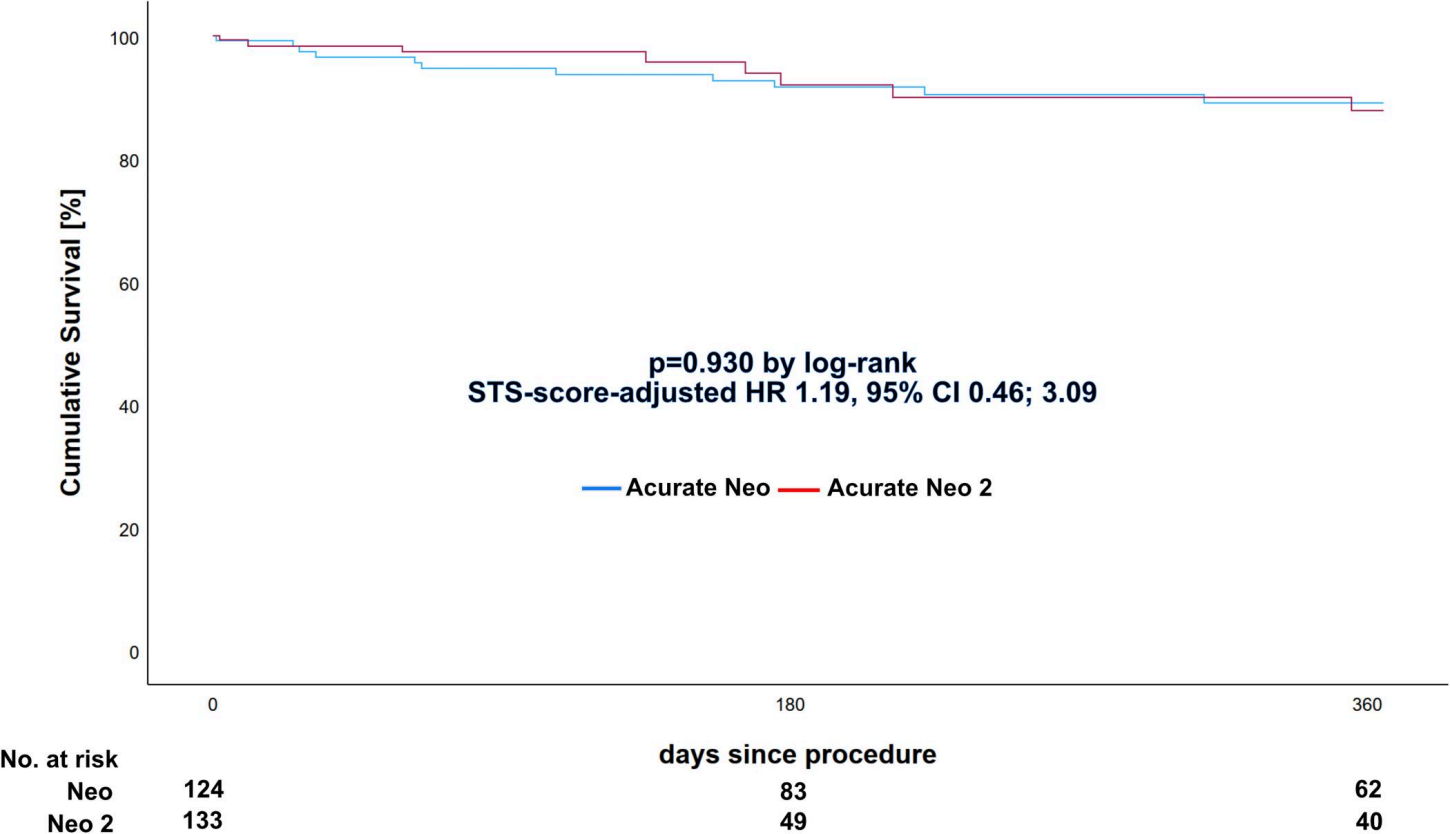
1‐Year survival comparing Acurate Neo with Acurate Neo 2. [Color figure can be viewed at wileyonlinelibrary.com]

The Kaplan−Meier estimated rates for freedom from the combined clinical endpoint death, stroke, myocardial infarction, and heart failure rehospitalization at 1 year were also comparable between groups (Acurate Neo 2: 81.2% [95% CI 72.0; 91.6] vs. Acurate Neo: 79.3% [95% CI 71.7; 87.8], *p* = 0.528). The STS‐score‐adjusted HR for the combined endpoint comparing Acurate Neo 2 versus Acurate Neo was 1.00 (95% CI 0.49; 2.04) (Figure 4).

**Figure 4:**
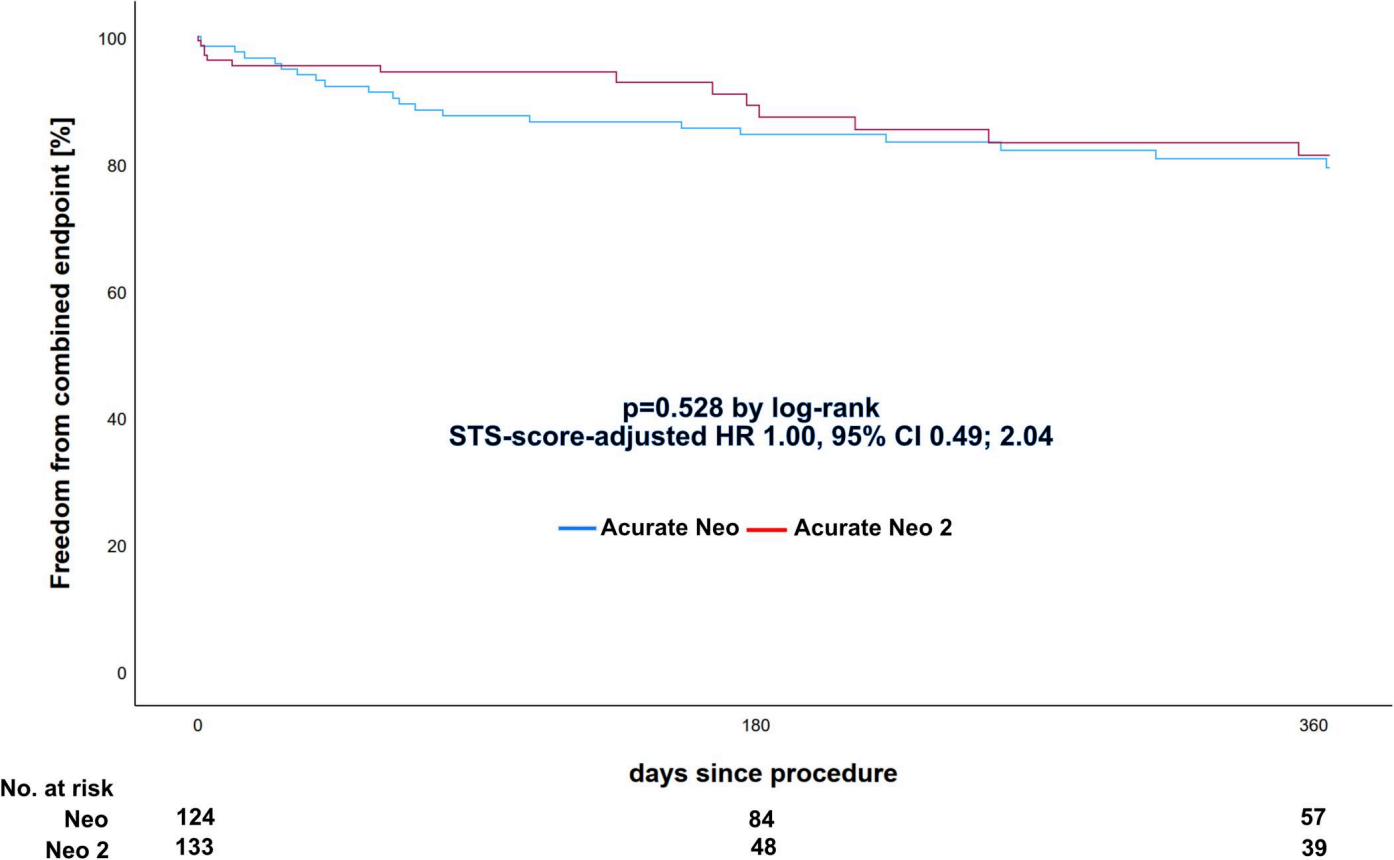
Freedom from all‐cause mortality, myocardial infarction, stroke, and heart failure rehospitalization comparing Acurate Neo with Acurate Neo 2 at 1‐year follow‐up. [Color figure can be viewed at wileyonlinelibrary.com]

At 3‐years, there was no difference between Acurate Neo 2 versus Acurate Neo with regard to survival (Acurate Neo 2: 65.8% [95% CI 52.6; 82.3] vs. Acurate Neo: 69.5% [95% CI 59.0; 81.9], *p* = 0.911) or freedom from the combined clinical endpoint (Acurate Neo 2: 56.9% [95% CI 44.3; 73.2] vs. Acurate Neo: 53.5% [95% CI 42.8; 66.9], *p* = 0.831) (Supporting Information S1: Figures S1 and 2).

### 3.6 Impact of Midframe Under‐Expansion

Midframe under‐expansion occurred in 13.3% of all patients. Despite higher rates of postdilatation, Acurate Neo had numerically higher rates of midframe under‐expansion compared to Acurate Neo 2 (17.8% vs. 9.9%, *p* = 0.071). Baseline and procedural characteristics, as well as outcomes comparing patients with and without midframe under‐expansion, are shown in Supporting Information S1: Tables 3−4. Patients with midframe under‐expansion had slightly higher mean gradients and more moderate PVL after implantation. The use of Acurate Neo 2 (OR 0.34, 95% CI 0.15; 0.79) and postdilatation (OR 0.14, 95% CI 0.06; 0.33) were independently associated with a lower risk of midframe under‐expansion (Supporting Information S1: Table 5).

Midframe under‐expansion was associated with a significantly lower freedom from the combined endpoint of death, stroke, myocardial infarction, and heart failure rehospitalization after 1 year (midframe under‐expansion: 65.2% [95% CI 49.5; 86.0] vs. no midframe under‐expansion: 84.7% [95% CI 78.8; 91.0], *p* = 0.007). The STS‐score‐ and Acurate Neo versus Neo 2‐adjusted HR for the combined endpoint comparing midframe under‐expansion versus no midframe under‐expansion was 2.33 (95% CI 1.10; 4.97) (Figure 5). There was no significant interaction for midframe under‐expansion in Acurate Neo and Acurate Neo 2 at 1‐year follow‐up (Supporting Information S1: Figure 3).

**Figure 5:**
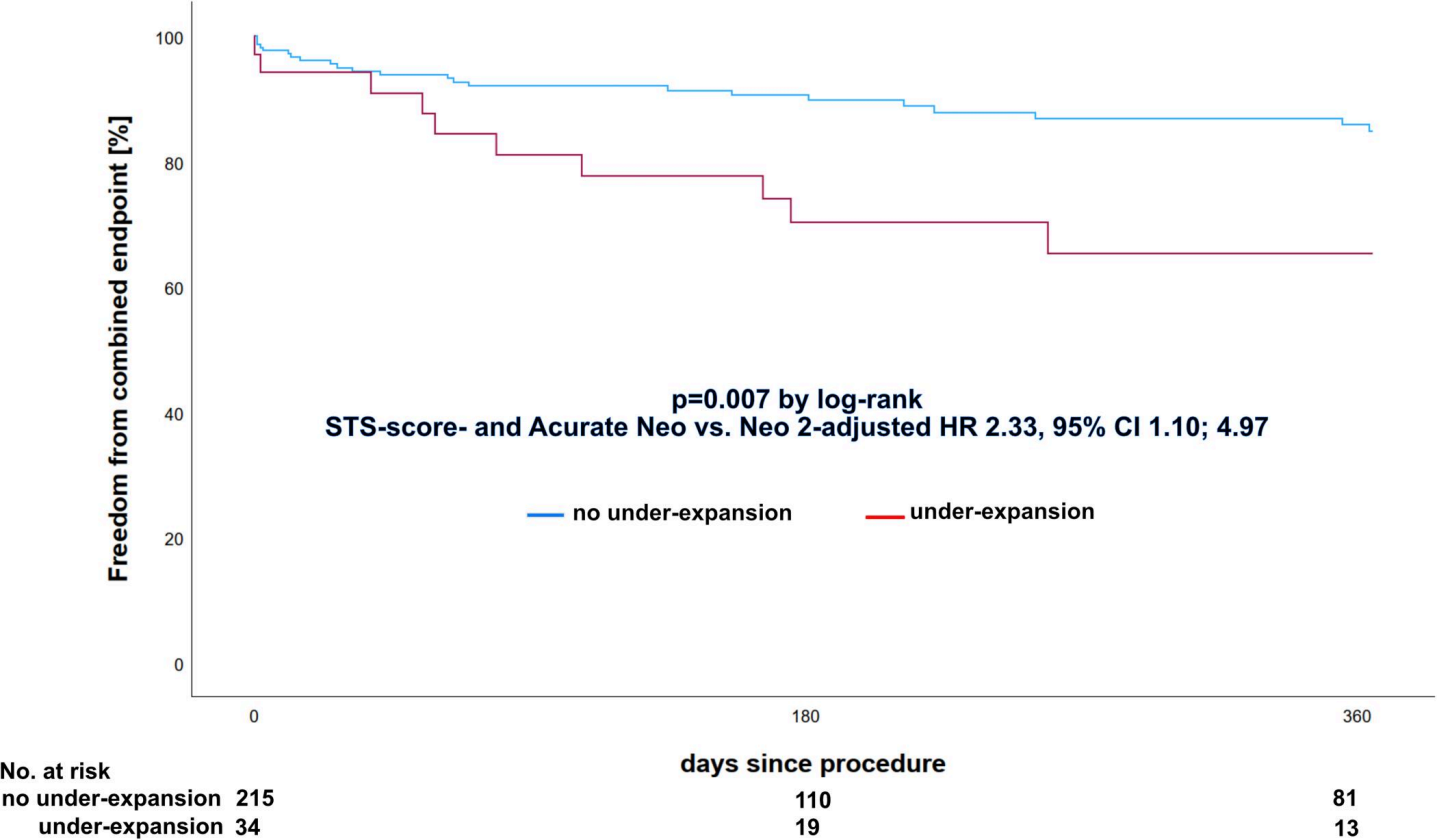
Freedom from all‐cause mortality, myocardial infarction, stroke, and heart failure rehospitalization comparing patients with midframe under‐expansion with no midframe under‐expansion. [Color figure can be viewed at wileyonlinelibrary.com]

After 3 years, the difference between the two groups was no longer statistically significant with regard to the combined clinical endpoint (midframe under‐expansion: 52.2% [95% CI 34.5; 78.9] vs. no midframe under‐expansion: 59.1% [95% CI 49.7; 70.2], *p* = 0.087) (Supporting Information S1: Figure 4). A landmark analysis, which began after 1 year of follow‐up, revealed that the difference occurred within the first year, after which the rates of the combined clinical endpoint were comparable (Supporting Information S1: Figure 4).

## 4 Discussion

This retrospective, single‐center analysis compared the performance of the Acurate Neo and Acurate Neo 2 transcatheter heart valves in patients undergoing TAVI. Acurate Neo 2 significantly improved clinical outcomes, particularly in terms of device success, early safety, and the reduction of PVL, compared to its predecessor, the Acurate Neo valve. Clinical endpoints were not different between groups at 1‐ and 3‐year of follow‐up. However, midframe under‐expansion occurred with both systems and was associated with worse clinical outcomes within the first year after implantation.

Our findings are consistent with previous studies [18] that highlighted the ability of the Acurate Neo 2 valve to reduce PVL. In a multicenter registry comparing the two generations [19], the Acurate Neo 2 reduced the incidence of moderate or severe PVL, which is corroborated by our finding that moderate PVL was nearly eliminated in the Acurate Neo 2 group. The lower rates of PVL observed with Acurate Neo 2 are clinically significant, as PVL is associated with increased long‐term mortality after TAVI [20, 21]. The addition of the pericardial sealing skirt in the Acurate Neo 2 valve design appeared to be highly effective in addressing one of the primary limitations of the original Acurate Neo valve. Importantly, the addition of the sealing skirt did not lead to an increased risk for new PPI. The reduction in PVL, combined with lower rates of major vascular complications, led to a higher device success with the use of Acurate Neo 2.

Our study also showed a significant improvement in early safety at 30 days with the Acurate Neo 2 compared to Acurate Neo. This is consistent with data from large registries, suggesting that the Acurate Neo 2 valve has a better safety profile due to lower rates of complications such as major bleeding and access site complications [22]. Life‐threatening or major bleeding was significantly lower in Acurate Neo 2 in our analysis. Although speculative, the reduced number of bleeding and vascular complications associated with Acurate Neo 2 may be attributed to a combination of treatment‐era‐related factors, including the redesigned expandable introducer with a low‐profile iSleeve, Boston Scientific (although iSleeve was introduced in our center in March 2018, only a small number of patients were treated with other sheaths), increased operator experience, and improved vascular access and complication management (e.g., systematic use of ultrasound‐guided puncture).

In terms of hemodynamic performance, both valves provided excellent outcomes, with similar, very low residual mean gradients (7 mmHg in both groups). This indicates that both the Acurate Neo and Neo 2 valves offer effective relief of aortic stenosis with good hemodynamic profiles post‐procedure. Further long‐term follow‐up studies are necessary to investigate whether these short‐term findings result in adequate long‐term performance and freedom from reintervention. Up to 3 years after treatment, no statistically significant difference in survival or freedom from the combined clinical endpoint of death, stroke, myocardial infarction, or rehospitalization due to heart failure was observed between groups. This finding is comparable to other European registry analyses [22].

We observed a substantial difference between the groups in the evaluated and commonly used risk prediction scores, with lower STS Scores in the Acurate Neo 2 group. This suggests a reduction of morbidity and mortality risk in the study population, which is potentially driven by the expanded indication for TAVI in younger and less sick patients. But it is also important to consider that the STS model is an adaptive risk stratification tool, and calculated risk scores may vary over time due to updates in the algorithm. These changes often reflect advancements in surgical techniques, device technology, and shifts in patient populations. Furthermore, during the study period, patient selection for TAVI has shifted to lower‐risk patients with growing evidence of safety and efficacy in these populations, and the renewal of the ESC Guideline on the management of valvular heart disease in 2021 credited this by expanding recommendations for transcatheter treatment [4]. Thereby, a decrease in predicted mortality by risk scores also reflects guideline‐directed therapy in this real‐world cohort.

The Acurate Neo 2 has been evaluated in the ACURATE IDE trial, in which it failed to demonstrate noninferiority [15]. Compared with commercial valves, the ACURATE Neo 2 resulted in significantly worse outcomes with respect to the primary endpoint of a composite of all‐cause mortality, all‐stroke, and rehospitalization at 1 year. A post hoc analysis revealed that midframe under‐expansion of the Acurate Neo 2 valve was associated with a higher risk of the composite primary endpoint [16]. The rate of midframe under‐expansion was 21.6% in the ACURATE IDE trial, which is substantially higher compared to our analysis (13.7% overall, 9.9% in the Acurate Neo 2 cohort) and to real‐world data of Acurate Neo 2 derived from other centers in Europe (13.9%) [23]. Initial midframe under‐expansion might be even higher, up to 57% in our cohort, but it can be safely and effectively treated by postdilatation. Abandoning postdilatation was the strongest predictor of final midframe under‐expansion. Inexperience with the Acurate transcatheter heart valve and a lack of awareness of under‐expansion despite acceptable hemodynamic results (PVL and gradient) may have contributed to the higher rate of under‐expansion in the ACURATE IDE trial. These factors may also explain the higher rates of under‐expansion in the Acurate Neo compared to the Acurate Neo 2 in our analysis. Regardless of Acurate Neo or Acurate Neo 2, midframe under‐expansion was associated with worse clinical outcome as indicated by a lower freedom of the composite clinical outcome after 1 year. This finding is very consistent to the results of the ACURATE IDE trial [16] and other real‐world experience [23] indicating that midframe under‐expansion is an important risk factor for worse clinical outcomes in the use of the Acurate transcatheter heart valve system. It appears to be effectively treatable by postdilatation but requires awareness and clear definitions assessed during the procedure.

Against this background and additional regulatory requirements made by CE authorities, Boston Scientific decided to discontinue the commercialization of the ACURATE product platform in all markets where ACURATE is commercially available (ww.bfarm.de, reference 97307925F‐FA). It remains unclear how to follow‐up with patients who have midframe under‐expansion, and whether it is clinically relevant to bring them back for postdilatation. Interestingly, in our 3‐year follow‐up, the rates of the combined clinical endpoint were no longer significantly different between patients with and without under‐expansion. Landmark analysis revealed that the difference occurred within the first year. After that, the rates of the combined clinical endpoint were comparable, indicating that midframe under‐expansion is particularly detrimental within the first year after implantation.

The results of the ACURATE IDE trial [15] are contrasted with those of the DOUBLE‐CHOICE trial [24]. The latter showed that the primary endpoints of all‐cause mortality, stroke, moderate‐to‐severe prosthetic valve regurgitation, and PPI occurred significantly less frequently in the Acurate Neo 2 group than in the Evolut group at 30 days (15.4% vs. 30.4%). This demonstrates the noninferiority of the Acurate Neo 2 in a well‐selected TAVI cohort.

Several limitations should be considered in this study. First, the retrospective design is inherently prone to selection bias, and unmeasured confounders may have influenced the outcomes. Second, the single‐center nature of the study limits the generalizability of our findings to broader patient populations. Third, while we demonstrated significant improvements with the Acurate Neo 2 valve, longer‐term follow‐up than 3 years is necessary to assess the durability and sustained clinical benefits of this newer valve design. Unfortunately, in our study cohort, echocardiographic follow‐up is limited, which precludes us from drawing meaningful conclusions. Additionally, despite our best efforts to control for baseline characteristics, the Acurate Neo 2 group had a lower STS score, which may have contributed to the observed differences in outcomes. As event rates, particularly for mortality, were low, adjustment was only performed for the STS score, which incorporates different baseline characteristics such as age, sex, and comorbidities. However, this should be interpreted as a partial adjustment only, as the low number of patients and events is a significant statistical limitation. Fourth, the definition of midframe under‐expansion may vary between trials, and which one remains the best one predicting outcome remains unclear.

## 5 Conclusion

In this single‐center, retrospective analysis, the Acurate Neo 2 valve demonstrated significant improvements in device success, early safety, and reduction of paravalvular regurgitation compared to the Acurate Neo valve. Midframe under‐expansion occurred with both generations of Acurate transcatheter heart valves and was associated with worse clinical outcomes in the first year after implantation. Postdilatation reduces the risk of midframe under‐expansion and may improve clinical outcomes. These findings need to be interpreted with caution due to the limitations of the non‐randomized, observational single‐center design.

## Funding

The authors have nothing to report.

## Conflicts of Interest

S.H. received speakersʹ honoraria and consulting fees from Edwards Lifesciences, Abbott, Cardiac Dimensions, Meril, and Boston Scientific, outside the submitted work. F.J.W. received personal fees from Abiomed/J&J Med Tech, Abbott, Asahi Intecc, Boston Scientific, Corvia, Insight Lifetech, Teleflex, ShockWave, SMT, outside the submitted work. A.L. received consultant and speaker honoraria from Abbott Laboratories, Abiomed, AstraZeneca, Bayer, Boehringer Ingelheim, Boston Scientific Corporation, Corvia, Daiichi Sankyo Europe GmbH, Edwards Lifesciences, Medtronic USA, Novartis, Pfizer, and stock options for Picardia, Transverse Medical Inc., outside the submitted work. Within the last 36 months, N.M. received a research and an educational grant from Abiomed to his institution, outside the submitted work; an educational grant from BostonScientific to his institution, outside the submitted work; received personal fees from Abbott, Abiomed, Amgen, B. Braun, Biotronik, Boehringer Ingelheim, Boston Scientific, Cordis, Edwards Lifesciences, Inari, Medtronic, Pfizer/BMS, Shockwave, and SIS Medical, outside the submitted work. The other authors declare no conflicts of interest.

## Supporting information


Supporting File

## Data Availability

The data that support the findings of this study are available from the corresponding author upon reasonable request.
