# Supplementary material for: Comparison Between the Acurate Neo and the Acurate Neo 2 Transcatheter Heart Valve System: Impact of Midframe Under‐Expansion
Source: Catheter Cardiovasc Interv. 2026 Sep 1;108(5):1784–94. doi: 10.1002/ccd.70839 (PMC13725193; doi:10.1002/ccd.70839)
Supplement: Supplementary file 1 [file CCD-108-1784-s001.docx]

**Supplemental Material**

**Comparison Between the Acurate Neo and the Acurate Neo 2 Transcatheter Heart Valve System - Impact of Midframe Under-Expansion**

**Supplemental Table 1.** Predictors of Device Success

**Supplemental Table 2.** Predictors of Early Safety

**Supplemental Table 3.** Baseline and Procedural Characteristics according to midframe versus no midframe under-expansion

**Supplemental Table 4.** Procedural Outcome according to midframe versus no midframe under-expansion

**Supplemental Table 5.** Predictors of midframe under-expansion

**Supplemental Figure 1.** 3-year survival comparing Acurate Neo with Acurate Neo 2

**Supplemental Figure 2.** Freedom from all-cause mortality, myocardial infarction, stroke, and heart failure rehospitalization comparing Acurate Neo with Acurate Neo 2 at 3-year follow-up

**Supplemental Figure 3.** Impact of midframe under-expansion in Acurate Neo and Acurate Neo 2 at 1-year follow-up. UE indicates under-expansion.

**Supplemental Figure 4.** Impact of midframe under-expansion in Acurate Neo and Acurate Neo 2 at 3-year follow-up (A) and Landmark analysis starting at day 366 (B).

**Supplemental Table 1.** Predictors of Device Success

|  | **Univariate** | | **Multivariate** | |
| --- | --- | --- | --- | --- |
|  | **OR (95%-CI)** | **P-value** | **OR (95%-CI)** | **P-value** |
| Acurate neo 2 vs. neo | 2.86 (1.14; 7.16) | 0.025 | 2.68 (1.04; 6.94) | 0.042 |
| Age [per 1-year increase] | 0.99 (0.89; 1.09) | 0.828 |  |  |
| Male Gender | 1.34 (0.54; 3.37) | 0.529 |  |  |
| Body Mass Index [per 1 kg/m² increase] | 1.03 (0.94; 1.13) | 0.503 |  |  |
| STS-Score [per 1% increase] | 0.94 (0.85; 1.04) | 0.230 |  |  |
| NYHA III/IV | 0.81 (0.34; 1.93) | 0.634 |  |  |
| CAD | 2.47 (1.04; 5.86) | 0.041 | 3.47 (1.36; 8.84) | 0.009 |
| Previous CABG | 0.73 (0.20; 2.64) | 0.632 |  |  |
| Prev. PC | 0.61 (0.21; 1.74) | 0.350 |  |  |
| Atrial fibrillation | 0.44 (0.19; 1.02) | 0.056 |  |  |
| Arterial Hypertension | 0.88 (0.11; 7.11) | 0.903 |  |  |
| Diabetes mellitus | 0.79 (0.34; 1.86) | 0.587 |  |  |
| Prev. stroke | 0.44 (0.14; 1.43) | 0.174 |  |  |
| PAD | 0.66 (0.18; 2.40) | 0.525 |  |  |
| COPD | 3.02 (0.39; 23.23) | 0.290 |  |  |
| CKD stage ≥3b | 0.49 (0.21; 1.15) | 0.100 |  |  |
| Immunosuppressive Therapy | 1.47 (0.19; 11.70) | 0.716 |  |  |
| LV-ejection fraction [per 1 %increase] | 1.01 (0.98; 1.04) | 0.596 |  |  |
| Mean gradient [per 1 mmHg increase] | 0.99 (0.97; 1.02) | 0.659 |  |  |
| Mitral regurgitation 2/3 | 0.29 (0.12; 0.69) | 0.005 | 0.25 (0.10; 0.62) | 0.003 |
| Valve size |  |  |  |  |
| Large (27 mm) | reference |  |  |  |
| Medium (25 mm) | 1.21 (0.43; 3.37) | 0.718 |  |  |
| Small (23 mm) | 0.82 (0.29; 2.30) | 0.704 |  |  |
| Predilatation | n.a. |  |  |  |
| Postdilatation | 0.52 (0.21; 1.29) | 0.157 |  |  |
| Perimeter derived annulus [per 1 mm increase] | 1.01 (0.80; 1.27) | 0.951 |  |  |
| Eccentricity Index | 0.97 (0.90; 1.05) | 0.473 |  |  |
| Cover-Index [%] | 1.02 (0.92; 1.14) | 0.673 |  |  |
| Calcium Score DLZ > vs < median | 0.57 (0.24; 1.35) | 0.203 |  |  |
| CFA (per 1 mm increase) | 1.49 (0.97; 2.27) | 0.068 |  |  |
| CFA calcification (none/mild vs. moderate/severe) | 1.39 (0.50; 3.89) | 0.526 |  |  |

OR indicates odds ratio; CI; confidence interval; STS, Society of Thoracic Surgery; NYHA, New York Heart Association; CAD, coronary artery disease; CABG, coronary artery bypass surgery; PCI, percutaneous coronary artery intervention; PAD, peripheral artery disease; COPD, chronic obstructive lung disease; CKD, chronic kidney disease; LV, left ventricular; LVOT, left ventricular outflow tract; STJ, sinotubular junction; LCA, left coronary artery; RCA, right coronary artery; DLZ, device landing zone; CFA, common femoral artery.

**Supplemental Table 2.** Predictors of Early Safety

|  | **Univariate** | | **Multivariate** | |
| --- | --- | --- | --- | --- |
|  | **OR (95%-CI)** | **P-value** | **OR (95%-CI)** | **P-value** |
| Acurate neo 2 vs. neo | 2.37 (1.24; 4.54) | 0.009 | 2.36 (1.20; 4.65) | 0.013 |
| Age [per 1-year increase] | 1.01 (0.93; 1.08) | 0.905 |  |  |
| Male Gender | 0.82 (0.43; 1.56) | 0.540 |  |  |
| Body Mass Index [per 1 kg/m² increase] | 0.94 (0.88; 0.99) | 0.026 | 0.94 (0.89; 1.00) | 0.066 |
| STS-Score [per 1% increase] | 0.96 (0.89; 1.04) | 0.360 |  |  |
| NYHA III/IV | 0.56 (0.29; 1.10) | 0.094 |  |  |
| CAD | 1.02 (0.55; 1.92) | 0.944 |  |  |
| Previous CABG | 0.57 (0.22; 1.45) | 0.236 |  |  |
| Prev. PCI | 0.42 (0.19; 0.91) | 0.029 | 0.48 (0.21; 1.09) | 0.080 |
| Atrial fibrillation | 0.57 (0.30; 1.07) | 0.081 |  |  |
| Arterial Hypertension | n.a. | n.a. |  |  |
| Diabetes mellitus | 0.43 (0.23; 0.81) | 0.009 | 0.54 (0.28; 1.05) | 0.070 |
| Prev. stroke | 1.63 (0.47; 5.73) | 0.445 |  |  |
| PAD | 0.83 (0.29; 2.37) | 0.733 |  |  |
| COPD | 0.45 (0.19; 1.06) | 0.067 |  |  |
| CKD stage ≥3b | 1.04 (0.54; 2.01) | 0.898 |  |  |
| Immunosuppressive Therapy | 0.94 (0.26; 3.46) | 0.924 |  |  |
| LV-ejection fraction [per 1 %increase] | 1.00 (0.98; 1.03) | 0.975 |  |  |
| Mean gradient [per 1 mmHg increase] | 1.02 (1.00; 1.04) | 0.099 |  |  |
| Mitral regurgitation 2/3 | 0.57 (0.28; 1.15) | 0.114 |  |  |
| Valve size |  |  |  |  |
| Large (27 mm) | reference |  |  |  |
| Medium (25 mm) | 1.74 (0.84; 3.61) | 0.134 |  |  |
| Small (23 mm) | 1.61 (0.73; 3.56) | 0.237 |  |  |
| Predilatation | 0.70 (0.08; 5.93) | 0.742 |  |  |
| Postdilatation | 1.10 (0.59; 2.06) | 0.762 |  |  |
| Perimeter derived annulus [per 1 mm increase] | 0.91 (0.77; 1.09) | 0.298 |  |  |
| Eccentricity Index | 1.00 (0.98; 1.01) | 0.718 |  |  |
| Cover-Index [%] | 1.00 (0.92; 1.09) | 0.986 |  |  |
| Calcium Score DLZ > vs < median | 0.95 (0.51; 1.77) | 0.874 |  |  |
| CFA (per 1 mm increase) | 0.96 (0.71; 1.30) | 0.781 |  |  |
| CFA calcification (none/mild vs. moderate/severe) | 0.68 (0.35; 1.33) | 0.261 |  |  |

OR indicates odds ratio; CI; confidence interval; STS, Society of Thoracic Surgery; NYHA, New York Heart Association; CAD, coronary artery disease; CABG, coronary artery bypass surgery; PCI, percutaneous coronary artery intervention; PAD, peripheral artery disease; COPD, chronic obstructive lung disease; CKD, chronic kidney disease; LV, left ventricular; LVOT, left ventricular outflow tract; STJ, sinotubular junction; LCA, left coronary artery; RCA, right coronary artery; DLZ, device landing zone; CFA, common femoral artery.

**Supplemental Table 3.** Baseline and Procedural Characteristics according to midframe versus no midframe under-expansion

|  | **Midframe Under-expansion**  **n=34** | **No Midframe Under-expansion**  **n=215** | **P-value** |
| --- | --- | --- | --- |
| Age [years] | 83 (81; 87) | 83 (80; 86) | 0.382 |
| Male Gender, n (%) | 11/34 (32.4) | 77/215 (35.8) | 0.695 |
| Body Mass Index [kg/m²] | 26.1 (23.5; 30.1) | 26.8 (24.0; 30.4) | 0.534 |
| STS-Score [%] | 4.1 (2.9; 6.4) | 3.5 (2.6; 5.7) | 0.095 |
| EuroScore II | 4.3 (2.1; 7.4) | 3.3 (2.2; 5.2) | 0.242 |
| NYHA III/IV, n (%) | 25/34 (73.5) | 117/211 (55.5) | 0.047 |
| CAD, n (%) | 20/34 (58.8) | 124/215 (57.7) | 0.900 |
| Previous CABG, n (%) | 5/34 (14.7) | 18/215 (8.4) | 0.217 |
| Prev. PCI, n (%) | 4/34 (11.8) | 31/215 (14.4) | 0.796 |
| Atrial fibrillation, n (%) | 12/34 (35.3) | 66/215 (30.7) | 0.591 |
| Arterial Hypertension, n (%) | 32/34 (94.1) | 205/215 (95.3) | 0.671 |
| Diabetes mellitus, n (%) | 9/34 (26.5) | 80/215 (37.2) | 0.225 |
| Prev. stroke, n (%) | 2/34 (5.9) | 21/215 (9.8) | 0.750 |
| PAD, n (%) | 5/34 (14.7) | 17/215 (7.9) | 0.197 |
| COPD, n (%) | 2/34 (5.9) | 26/215 (12.1) | 0.389 |
| CKD stage ≥3b, n (%) | 13/33 (39.4) | 73/213 (34.3) | 0.566 |
| Immunosuppressive Therapy, n (%) | 3/34 (8.8) | 12/215 (5.6) | 0.439 |
| LV-ejection fraction [%] | 55 (41; 65)  N=34 | 58 (50; 64)  N=208 | 0.697 |
| Aortic valve area [cm²] | 0.7 (0.5; 0.8)  N=34 | 0.7 (0.6; 0.8)  N=211 | 0.768 |
| Mean gradient [mmHg] | 37 (29; 44)  N=34 | 39 (27; 47)  N=208 | 0.803 |
| Mitral regurgitation 2/3, n (%) | 10/34 (29.4) | 43/211 (20.4) | 0.235 |
| Indication |  |  |  |
| Native valve | 33/34 (97.1) | 215/215 (100) | 0.137 |
| valve-in-valve | 1/34 (2.9) | 0/215 (0) |  |
| Valve size |  |  | 0.577 |
| Large (27 mm) | 12/34 (35.3) | 70/215 (32.6) |  |
| Medium (25 mm) | 11/34 (32.4) | 89/215 (41.4) |  |
| Small (23 mm) | 11/34 (32.4) | 56/215 (26.0) |  |
| Predilatation, n (%) | 31/34 (91.2) | 210/214 (98.1) | 0.056 |
| Predilatation undersizing, [mm] | 1.2 (0.2; 1.7) | 1.3 (0.7; 2.0) | 0.209 |
| Postdilatation, n (%) | 11/34 (32.4) | 162/215 (75.3) | <0.001 |
| Postdilatation undersizing, [mm] | 1.4 (0.2; 2.3) | 1.0 (0.3; 1.8) | 0.315 |
| Perimeter derived annulus [mm] | 23.1 (21.5; 24.4) | 23.2 (22.1; 24.6) | 0.333 |
| Perimeter derived LVOT [mm] | 23.5 (21.5; 24.9) | 23.5 (22.2; 25.2) | 0.375 |
| Perimeter derived STJ [mm] | 27.2 (24.9; 29.2) | 27.5 (25.8; 29.4) | 0.259 |
| Height STJ [mm] | 21.8 (18.9; 22.6) | 21.6 (20.1; 23.1) | 0.237 |
| Height LCA [mm] | 13.1 (11.1; 15.1) | 13.2 (11.3; 15.3) | 0.616 |
| Height RCA [mm] | 16.5 (14.8; 18.7) | 16.6 (15.1; 18.3) | 0.952 |
| Eccentricity annulus | 1.32 (1.24; 1.41) | 1.33 (1.27; 1.39) | 0.745 |
| Cover-Index [%] | 8.3 (6.0; 10.9) | 7.4 (4.4; 9.6) | 0.080 |
| Calcium Score DLZ [AU] | 1766 (704; 5152) | 2433 (650; 6616) | 0.398 |
| Calcium Score Leaflets [AU] | 1742 (652; 4168) | 2305 (637; 5732) | 0.415 |
| Calcium Score LVOT [AU] | 82 (27; 614) | 1464 (8; 878) | 0.662 |
| Cusp symmetry, n (%) | 21/34 (61.8) | 97/215 (45.1) | 0.189 |
| CFA at access site [mm] | 8.1 (7.3; 8.8) | 8.2 (7.5; 8.9) | 0.526 |
| Moderate/severe calcification CFA, n (%) | 6/34 (17.6) | 58/213 (27.2) | 0.236 |

Variables are expressed as numbers and frequencies or median with 25^th^ and 75^th^ quartile. STS indicates Society of Thoracic Surgery; NYHA, New York Heart Association; CAD, coronary artery disease; CABG, coronary artery bypass surgery; PCI, percutaneous coronary artery intervention; PAD, peripheral artery disease; COPD, chronic obstructive lung disease; CKD, chronic kidney disease; LV, left ventricular; LVOT, left ventricular outflow tract; STJ, sinotubular junction; LCA, left coronary artery; RCA, right coronary artery; DLZ, device landing zone; CFA, common femoral artery.

**Supplemental Table 4.** Procedural Outcome according to midframe versus no midframe under-expansion

|  | **Midframe Underexpansion**  **n=34** | **No Midframe Underexpansion**  **n=215** | **P-value** |
| --- | --- | --- | --- |
| Technical success, n (%) | 33/34 (97.1) | 210/215 (97.7) | 0.590 |
| Device success, n (%) | 29/34 (85.3) | 198/215 (92.1) | 0.197 |
| Early safety, n (%) | 29/34 (85.3) | 174/215 (80.9) | 0.542 |
| 30-day mortality, n (%) | 1/34 (2.9) | 4/215 (1.9) | 0.523 |
| Myocardial infarction, n (%) | 0/33 (0) | 1/205 (0.5) | 1.000 |
| Stroke, n (%) | 0/33 (0) | 6/206 (2.9) | 1.000 |
| Life-threatening/major Bleeding, n (%) | 2/34 (5.9) | 11/215 (5.1) | 0.693 |
| Major access site complication, n (%) | 1/34 (2.9) | 4/215 (1.9) | 0.523 |
| Renal Failure, n (%) | 5/34 (14.7) | 20/215 (9.3) | 0.355 |
| New PPM/ICD, n (%) | 2/26 (7.7) | 15/177 (8.5) | 1.000 |
| Residual AR |  |  | 0.031 |
| No AR | 14/34 (41.2) | 69/215 (32.1) |  |
| Mild AR | 17/34 (50.0) | 142/215 (66.0) |  |
| Moderate AR | 3/34 (8.8) | 4/215 (1.9) |  |
| Residual mean gradient [mmHg] | 8 (6; 11) | 6 (4; 9) | 0.023 |

Variables are expressed as numbers and frequencies or median with 25^th^ and 75^th^ quartile. ICD indicates implantable cardioverter defibrillator, PPM, permanent pacemaker, AR, aortic regurgitation.

**Supplemental Table 5.** Predictors of midframe under-expansion

|  | **Univariate** | | **Multivariate** | |
| --- | --- | --- | --- | --- |
|  | **OR (95%-CI)** | **P-value** | **OR (95%-CI)** | **P-value** |
| Acurate neo 2 vs. neo | 0.51 (0.24; 1.07) | 0.074 | 0.34 (0.15; 0.79) | 0.012 |
| Age [per 1-year increase] | 1.03 (0.95; 1.13) | 0.465 |  |  |
| Male Gender | 0.86 (0.40; 1.85) | 0.695 |  |  |
| Body Mass Index [per 1 kg/m² increase] | 0.98 (0.91; 1.05) | 0.575 |  |  |
| STS-Score [per 1% increase] | 1.06 (0.97; 1.16) | 0.183 |  |  |
| NYHA III/IV | 2.23 (0.99; 5.01) | 0.052 |  |  |
| CAD | 1.05 (0.50; 2.19) | 0.900 |  |  |
| Previous CABG | 1.89 (0.65; 5.47) | 0.242 |  |  |
| Prev. PC | 0.79 (0.26; 2.40) | 0.680 |  |  |
| Atrial fibrillation | 1.23 (0.58; 2.64) | 0.592 |  |  |
| Arterial Hypertension | 0.78 (0.16; 3.73) | 0.756 |  |  |
| Diabetes mellitus | 0.61 (0.27; 1.37) | 0.228 |  |  |
| Prev. stroke | 0.58 (0.13; 2.58) | 0.472 |  |  |
| PAD | 2.01 (0.69; 5.86) | 0.202 |  |  |
| COPD | 0.45 (0.10; 2.01) | 0.298 |  |  |
| CKD stage ≥3b | 1.25 (0.59; 2.65) | 0.566 |  |  |
| Immunosuppressive Therapy | 1.64 (0.44; 6.13) | 0.464 |  |  |
| LV-ejection fraction [per 1 %increase] | 1.00 (0.97; 1.03) | 0.849 |  |  |
| Mean gradient [per 1 mmHg increase] | 1.00 (0.98; 1.02) | 0.906 |  |  |
| Mitral regurgitation 2/3 | 1.63 (0.72; 3.66) | 0.238 |  |  |
| Valve size |  |  |  |  |
| Large (27 mm) | reference |  |  |  |
| Medium (25 mm) | 0.72 (0.30; 1.73) | 0.464 |  |  |
| Small (23 mm) | 1.15 (0.47; 2.79) | 0.764 |  |  |
| Predilatation | 0.20 (0.04; 0.92) | 0.039 | 0.44 (0.07; 2.76) | 0.383 |
| Postdilatation | 0.16 (0.07; 0.34) | <0.001 | 0.14 (0.06; 0.33) | <0.001 |
| Perimeter derived annulus [per 1 mm increase] | 0.87 (0.71; 1.06 | 0.159 |  |  |
| Eccentricity Index | 1.10 (0.06; 20.5) | 0.948 |  |  |
| Cover-Index [per 1 % increase] | 1.12 (1.02; 1.23) | 0.021 | 1.09 (0.98; 1.21) | 0.102 |
| Calcium Score DLZ > vs < median | 0.74 (0.36; 1.56) | 0.433 |  |  |
| DLZ Calcification (per 100 AU increase) | 1.01 (0.99; 1.02) | 0.463 |  |  |

OR indicates odds ratio; CI; confidence interval; STS, Society of Thoracic Surgery; NYHA, New York Heart Association; CAD, coronary artery disease; CABG, coronary artery bypass surgery; PCI, percutaneous coronary artery intervention; PAD, peripheral artery disease; COPD, chronic obstructive lung disease; CKD, chronic kidney disease; LV, left ventricular; LVOT, left ventricular outflow tract; STJ, sinotubular junction; LCA, left coronary artery; RCA, right coronary artery; DLZ, device landing zone.

**Supplemental Figure 1.** 3-year survival comparing Acurate Neo with Acurate Neo 2

**Supplemental Figure 2.** Freedom from all-cause mortality, myocardial infarction, stroke, and heart failure rehospitalization comparing Acurate Neo with Acurate Neo 2 at 3-year follow-up

**Supplemental Figure 3.** Impact of midframe under-expansion in Acurate Neo and Acurate Neo 2. UE indicates under-expansion.

**Supplemental Figure 4.** Impact of midframe under-expansion in Acurate Neo and Acurate Neo 2 at 3-year follow-up (A) and Landmark analysis starting at day 366 (B).
